# The complete genomic sequence of the type strain *Brochothrix thermosphacta* DSM 20171 highlights a diversity of prophages in this species

**DOI:** 10.1128/mra.00239-24

**Published:** 2024-07-02

**Authors:** Loic Gingras, Laurie C. Piché, Linda Saucier, Antony T. Vincent

**Affiliations:** 1 Département des Sciences Animales, Faculté des Sciences de l'Agriculture et de l'Alimentation, Université Laval, Quebec City, Quebec, Canada; 2 Institut de Biologie Intégrative et des Systèmes, Pavillon Charles-Eugène-Marchand, Université Laval, Quebec City, Quebec, Canada; 3 Institut sur la Nutrition et les Aliments Fonctionnels, Faculté des Sciences de L'agriculture et de l'Alimentation, Université Laval, Quebec City, Quebec, Canada; 4 Centre de recherche en infectiologie porcine et avicole, Faculté de médecine vétérinaire, Université de Montréal, Saint-Hyacinthe, Quebec, Canada; University of Maryland School of Medicine, Baltimore, Maryland, USA

**Keywords:** *Brochothrix thermosphacta*, genomic islands, Illumina, Nanopore, prophages, type strain

## Abstract

The bacterium *Brochothrix thermosphacta* is a known muscle food spoiler. Here, the complete genome sequence of the *B. thermosphacta* type strain, DSM 20171, is reported. Prediction of prophages and genomic islands reveals an unsuspected diversity in this bacterial species that deserves further investigation.

## ANNOUNCEMENT

*Brochothrix thermosphacta* is a species in the *Listeriaceae* family (1). It is a Gram-positive, facultatively anaerobic, and psychrotrophic bacteria (1). Furthermore, it is recognized as a meat and other muscle food spoiler (2).

Having complete genome sequences of type strains is important to decipher bacterial evolution (3). The type strain *B. thermosphacta* DSM 20171 was isolated from fresh pork sausage (4). Sequences of this type strain are currently available, but the assemblies are in draft condition, making it difficult to use these sequences to study genome architecture and mobile DNA elements. Here, the chromosome sequence of the type strain *B. thermosphacta* DSM 20171 has been closed using Nanopore and Illumina technologies.

The type strain *B. thermosphacta* DSM 20171 was obtained from the DSMZ collection (Braunschweig, Germany) and was previously deposited by W.L. Sulzbacher. The total DNA was extracted with QIAamp PowerFecal Pro DNA Kit (QIAGEN, Toronto, ON, Canada) from a culture grown on Heart Infusion Broth agar medium and incubated for 24 h at 25°C following the manufacturer’s instructions. The extracted DNA was quantified by fluorescence using the PicoGreen kit (Invitrogen, Waltham, MA, USA) and sequenced on an Illumina NextSeq2000 (2 × 150 bp) and a Nanopore PromethION by Plasmidsaurus (Eugene, OR, USA). The Illumina library was prepared using the SeqWell ExpressPlex 96 library prep kit, while the Nanopore library was prepared using v14 library prep chemistry without fragmentation or size selection and sequenced on an R10.4.1 flow cell. Base calling was carried out for Nanopore reads using Dorado version 7.1.4 (5) on super-accurate mode. Illumina sequencing reads were filtered with Fastp version 0.23.2 (6) and those from Nanopore were filtered using Filtlong version 0.2.1 (7) by keeping the best 90% of reads above 1,000 bp or until only 500 Mbp remained. Read quality was verified with FastQC version 0.12.1 (8). After filtration, 10.87 M Illumina reads were retained, totaling 1.71 Gb (90% > Q20, 82% > Q30). For Nanopore reads, 40,970 reads (N50 ~8 kb) were retained, totaling 0.27 Gb. A hybrid assembly was done using Unicycler version 0.5.0 (9). As the sequence was considered circular by Unicycler version 0.5.0, redundant ends were removed, and it was linearized to the *dnaA* gene using the same tool. Prophages and genomic islands were predicted using Phastest (10) and Islandviewer version 4 (11), respectively. Default parameters were used for all software unless otherwise specified.

**Fig 1:**
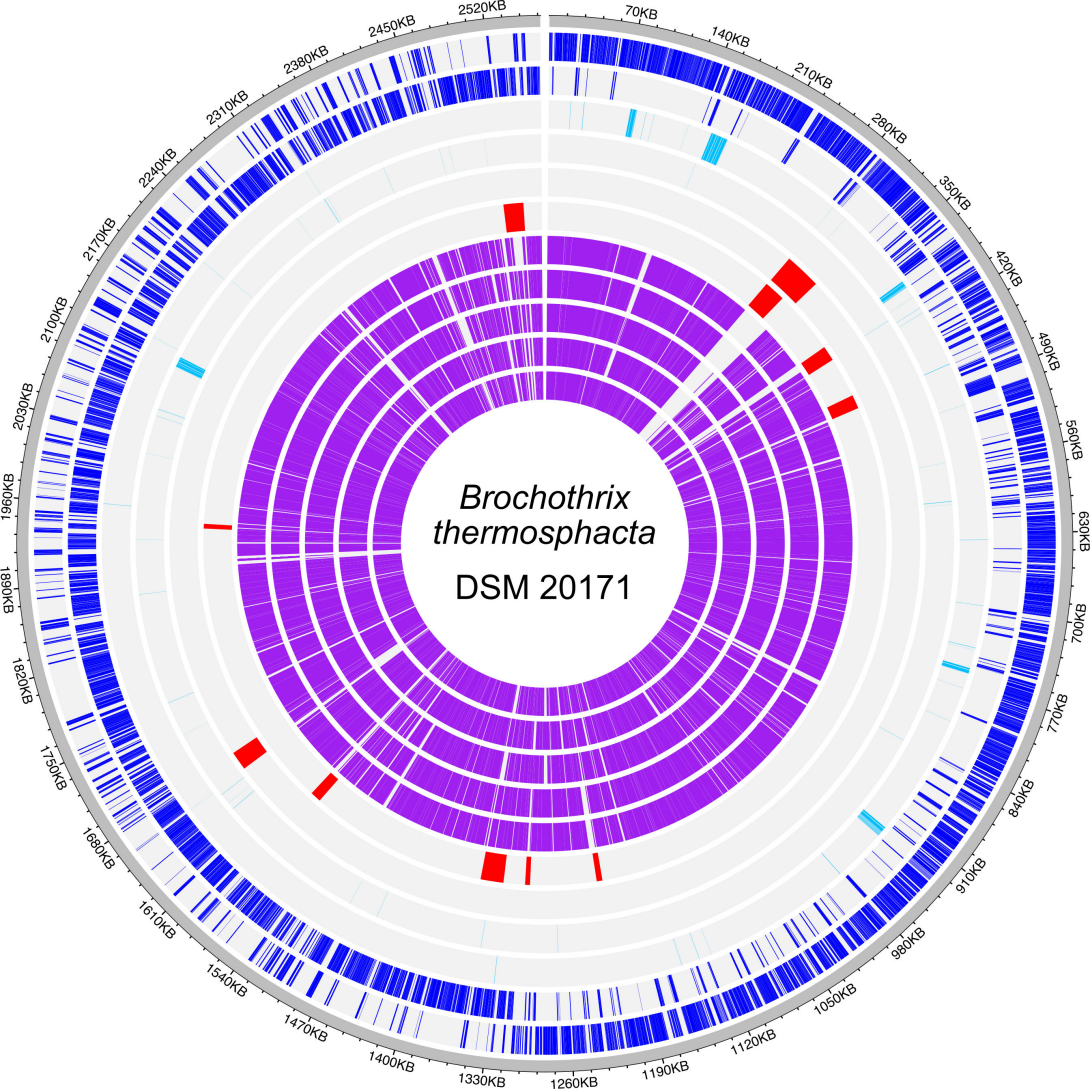
Chromosome sequence map of *B. thermosphacta* DSM 20171. From outside to inside: genes encoding coding sequences (CDSs) on the leading strand (dark blue), genes encoding CDSs on the lagging strand (dark blue), genes encoding RNAs on the leading strand (light blue), genes encoding RNAs on the lagging strand (light blue), prophage prediction (red), genomic islands prediction (red), and homologous regions (purple) between the type strain DSM 20171 and strains BI (CP023483.1), BII (CP023643.1), CD 337 (LT993737.1), TMW 2.1564 (CP016839.1), and TMW 2.1572 (CP016841.1) available in GenBank. The homologous regions were defined by CONTIGuator version 2.7 (13) and the figure was created with ShinyCircos version 2.0 (14).

A single circular sequence of 2,564,037 bp with a G + C percentage of 36.58 was obtained. The mean coverage of the sequence was 660×. The annotation by NCBI with the PGAP tool version 6.6 (12) revealed 2,375 genes encoding proteins and 86 genes encoding tRNAs. No plasmids were found. The complete sequence of the *B. thermosphacta* type strain allowed its comparison with five other complete genome sequences of *B. thermosphacta* listed in GenBank (Fig. 1). The prediction of prophages and genomic islands revealed diversity between the *B. thermosphacta* genome sequences analysed, as shown by the absence of homology within these regions for several strains. This demonstrates the value of studying the mobilome in this bacterium.

## Data Availability

The complete chromosome sequence of *B. thermosphacta* DSM 20171 has been deposited in DDBJ/ENA/GenBank under the accession no. CP145608. Illumina and Nanopore sequencing reads were deposited in the Sequence Read Archive database under accession numbers SRX23715081 and SRX23715082, respectively.
